# Navigating performativity via stage directions expressing emotion: a case study of the English-Chinese drama translation of *Lady Windermere’s Fan*

**DOI:** 10.3389/fpsyg.2026.1900774

**Published:** 2026-09-15

**Authors:** Wenshuang Li, Qiurong Zhao

**Affiliations:** 1 School of Foreign Studies, Nankai University, Tianjin, China; 2 School of Foreign Studies, University of Science and Technology Beijing, Beijing, China

**Keywords:** affective pragmatics, performativity, stage directions, drama translation, *Lady Windermere’s Fan*

## Abstract

The affective pragmatic investigation of stage directions offers a valuable lens for how emotional expression activates the performativity of playtexts, as exemplified by the landmark success of Hong Shen’s English-Chinese translation of *Lady Windermere’s Fan*. This study integrates corpus-based quantitative analysis with affective pragmatic interpretation to examine Hong’s translation in comparison with two other representative Chinese versions by Shen Xingren and Yu Guangzhong. The findings reveal that Hong’s translation systematically reconfigures emotion in stage directions through addition, omission and adaptation (including typological shift and content change). These strategies collectively produce increased emotional density, intensified conflict with a shift toward neutrality, polarized emotional intensity, and amplified emotional fluctuations. Underpinning this reconfiguration are three communicative moves derived from affective pragmatics: declaratives make characters’ psychology explicit, imperatives embody verbal orders into gestic cues, and commissives drive the plot by anchoring characters’ future actions. The study argues that stage directions expressing emotions constitute a pre-performing space wherein the translator negotiates between source-text fidelity and stage-oriented performativity, thereby positioning the drama translator as an active co-constructor for future performance. The findings underscore the significance of sentiment analysis and affective pragmatics in investigating drama translation.

## 1 Introduction

Oscar Wilde’s classic play, *Lady Windermere’s Fan*, was among the first Western dramas introduced to China during the May Fourth Movement. To date, the play has been translated into Chinese more than 8 times. Among these, three versions stand out for their exceptional scholarly and literary merits. Shen Xingren pioneered the introduction of the play in vernacular Chinese; Yu Guangzhong’s translation, guided by the principle of pleasing the reader’s eyes, audience’s ear, and actor’s tongue (Yu, 2000, p. 151), attends to dialogue rhythm on a literal basis but leaves stage directions rigidly literal and the play itself unstaged (Zhao and Li, 2025, p. 123); Hong Shen’s 1923 version,^1^ by contrast, distinguishes itself from its predecessors.

Hong Shen’s distinctiveness lies in his motivation. The 1920s marked a crucial transitional phase for Chinese drama. Tian Han, Guo Moruo, Cao Yu, and other bilingual intellectuals with cross-cultural perspectives engaged actively in theatrical pursuits, yielding substantial translated and adapted works. Hong criticized the word-by-word approach of his predecessors, which was unsuitable for the stage and failed to capture nuanced subtext. The interpretation of psychology must be rendered through the synergy of dialogue and onstage action” (Hong, 1988, p. 466). This critical stance drove him from the outset to produce a text oriented toward performance on stage. Such motivation directly shaped his textual strategies: he employed techniques such as localization (An, 2008), cultural filtering (Liang and Zhang, 2013), and ethical adaptation (Liu, 2021) to foreground dialogue rhythm and its potential for transforming into action by incorporating detailed stage directions (Zhao and Li, 2025, p. 119). These textual choices were merely from theoretical reflection, but grounded in actual theatrical practice, as he involved himself in every phase of production as translator, director, and supporting actor.

Hong’s adaptation proved to be a stage success: the production was premiered in 1924 by the Shanghai Stage Society,^2^ and received widespread acclaim (Ying, 1959, p. 3). Performances directed by him were lauded for natural dialogue and actions (Zhou, 1924, p. 18) and tremendous emotional power (Qing, 1924, p. 20), a feat achieved through a dual-track reconfiguration of dialogue and stage directions. Its cultural impact was further solidified through the adaptation for film in 1928 and later served as an authoritative reference for subsequent performances by the Shanghai Huju Opera Troupe. The production demonstrated enduring stage viability and cultural resonance. The combination of his critical motivation, textual adaptations, and hands-on stage involvement together make his translation exemplary for investigating the performability of drama, a quality evident both in its dialogue and stage directions.

Stage directions (didascalia in Latin) indicate the psychological or physical state of the characters, and the frame of the action (Pavis, 1998, p. 355–357). Historically, stage directions did not truly appear until the early eighteenth century, pioneered by authors such as Houdar de la Motte in his *Inés de Castro* (1723) and *Marivaux*; they became general practice with naturalistic theater (Pavis, 1998, p. 356). Functioning as a commentary for future performances, stage directions serve as a persistent mediator between text and stage. They constitute a semiotic system that transforms abstract emotions, felt directly as bodily states, into perceptible, embodied signs (Peirce, 1960, p. 115–116). Therefore, how emotion is reconfigured within the translation of stage directions is pivotal to a playtext’s performability, a factor that largely accounts for the historic success of Hong’s translation. While dialogue articulates explicit conflict, stage directions crystallize underlying emotions into a tangible stage presence. This creates a “pre-performing space” (Pavis, 1998, p. 357) that serves as a liminal space where translators can transcend traditional notions of fidelity and engage in creative rewriting. Ultimately, this underscores the ontological status of playtexts as incomplete (Ubersfeld, 1999, p. xvi), awaiting completion through performance.

Despite the established significance of stage directions, scholarly inquiry into the performability of Hong Shen’s English-to-Chinese translation has remained disproportionately centered on dialogue. This critical oversight reflects a broader lacuna in drama translation studies, where stage directions are frequently relegated to a secondary status, while stage directions function as pivotal signs within the text-performance continuum. And translators, as active co-constructors of signs, should employ dynamic strategies that negotiate source-language norms and target-language theatrical requirements to facilitate cross-cultural emotional reconfiguration and then enhance performability. Building on this assertion, this study seeks to examine how a successful drama translation reconfigures stage directions expressing emotions and then adapts them more effectively for performance.

## 2 Rethinking drama translation: from linguistic paradigms to stage performance

Drama translation studies have evolved from a narrow linguistic focus toward a comprehensive, multi-dimensional paradigm. While early structuralist inquiries by the Prague School in the 1930s laid groundwork by analyzing the formal dynamics of meaning production (Pavis, 1998, p. 326), it was the shift in the 1970s that fundamentally redefined the field. Scholars such as Pavis and Ubersfeld transcended linguistic paradigms, arguing that playtexts cannot be considered identical to texts written to be read because the process of writing involves a consideration of the performance dimension (Bassnett, 1991, p. 110).

In the 21st century, this evolution has culminated in a definitive turn toward performance. Driven by the proliferation of streaming technologies and digital subtitling or surtitling, translation is now defined as a link in the transformation of stage signs, and its function has extended from textual conversion to a systemic intervention in stage production (Heylen, 1993; Leppihalme, 2000). This concern crystallizes in the notion of performability, a term broadly defined as the text’s pragmatic adaptability to the stage (Glynn and Hadley, 2021), while also used to describe the indescribable, supposedly existent gestic text within the written (Bassnett, 1991, p. 102).

Given its conceptual divergence, three main areas of enquiry are identified under the umbrella term of performability studies. The first examines drama and theater as practiced in performance. The second, developed out of the work by Schechner and Turner (1985), describes cultural rituals through the lens of theatrical metaphors and terminology. The third investigates how language functions via the concepts of the performative (Searle, 1969) and performativity (Butler, 1997), a concept rooted in Austin (1962) speech act theory. While performativity emphasizes the capacity of language to operate as a form of social action, performability encompasses both the linguistic attributes that facilitate stage transitions and the power dynamics between agents, norms and cultures (Yang, 2021, p. 886).

The three trajectories outlined above find their common ground in Munday et al., 2022, p. 71) continuum of drama translation, a collaboration that operates in drama translation: interlingual (dramatic translation), intra-lingual (script as spoken on stage), inter-semiotic (script turned into performance), and dependence of performance on previous performances, on stage or in other media. Within this continuum, the interlingual text-centered phase is fundamental. This is also evidenced by Hong Shen’s translation, which served as an authoritative source text for subsequent adaptations for performance. Stage directions, in this light, constitute the dialectical hinge between these phases: they are at once textual elements and performative catalysts, transforming semantic content into embodied action and thus integrating such a continuum into a unified theatrical event.

Current research on performativity, though often nested within performability studies, remains largely confined to formal textual features. This emphasis includes lexical features, such as modal particles and slang (Ren et al., 2014), idiosyncratic word choices (Glynn and Hadley, 2021), and other stylistic features, such as prosodic phonology (Ren et al., 2022) and rhetoric and poetics (Wu, 2024). However, the purely linguistic approach fails to account for performativity, the very quality that distinguishes drama from other literary genres. More critically, the affective dimension, which enables stage directions to exert illocutionary effects in performance, has received scant attention.

Aristotle (1996, p. 12) posits that the very existence of drama hinges on emotional arousal. Theories of drama further radicalize this view by emphasizing liberating drama from its textual origins and presenting a more pure and heightened form of emotion and physicality (Artaud, 1964, p. 146). In drama translation, translators likewise seek to recreate in the audience an emotional response comparable to that evoked by the original play (Brodie and Cole, 2017, p. 92). To operationalize such affective dynamics, *Dictionary of Affect in Language* has been used to quantify the emotional undertones, specifically pleasantness, activation, and imagery of translators’ lexical choices, offering an affective lens for the analysis of translator style (Whissell, 2011). Emotional words have also been drawn to construct emotional valence curves for examining differences among drama translations (Ding, 2024).

Existing studies rarely examine affective pragmatics as performative links between text and performance, instead reducing it to mere stylistic features. This gap widens when considering the functional divide between dialogue and stage directions. While dialogue yields subjective, polysemic emotions, stage directions secure explicit affective anchors instrumental for theatrical staging. Specifically, when translators like Hong Shen are also directors or performers, their rendering of stage directions visualizes the rehearsal process, opening up an avenue for analyzing performativity, namely, how affective cues in stage directions mediate the responses of readers, actors, and directors.

## 3 How to do things with emotional expressions?

Navigating performativity through emotion-laden stage directions ultimately hinges on a foundational question: how do emotional expressions communicate? In the field of affective science, there has long been a theoretical divergence regarding the nature and function of emotional expressions. The Ekman (1992) basic emotion view maintains that emotional expressions are reflexive outputs of basic affect programs, such as anger, fear, sadness, happiness, disgust, and surprise, driven by dedicated neural circuits. According to this view, each basic emotion is linked to a specific facial expression pattern that is recognized cross-culturally in a universal manner. This constitutes the foundation of sentiment analysis in computational linguistics.

Fridlund (1994) behavioral ecology view completely rejects the concept of emotional expression and replaces it with display behaviors, arguing that facial and bodily movements are highly context-dependent social tools whose primary function is not to convey internal feelings but to serve social motives, that is, to declare intentions, issue requests, and influence the behavior of others. Fridlund’s critique goes to an extreme: he entirely dismisses emotion as an explanatory construct, severing the probabilistic correlations between emotional states and social motives.

These two paradigms are sharply opposed by treating expressing emotions and conveying social information as mutually exclusive alternatives. Yet the very process of expressing emotions via language itself also conveys social information. In other words, emotional expressions are means not only of expressing what’s inside but also of directing other people’s behavior, of representing what the world is like, and of committing to future courses of action (Scarantino, 2017, p. 165). This continuum is analogous to the semiosis in Peirce’s semiotics.^3^ For instance, “angrily” in playtexts not only expresses the character’s emotion, but also instructs the actor to enact aggressive physicality, signals the dramatic crisis to the audience, and sets in motion a trajectory of escalating conflict.

To further clarify the continuum of emotional expression, Scarantino (2017) first draws upon Austin, (1962) speech act theory, which distinguishes three types of acts performed in utterances: the locutionary act (the act of saying something), the illocutionary act (what one does in saying it), and the perlocutionary act (what one achieves by saying it). Scarantino then shifts the focus from linguistic utterances to non-verbals, by reinterpreting the illocutionary act as communicative moves performed via emotional expressions. Furthermore, drawing on Searle, 1979) fivefold taxonomy of illocutionary acts, Scarantino (2017: 173) proposes four categories of communicative moves for emotional expression:

Expressives (correspond to Searle’s expressives) convey emotions;

Imperatives (correspond to Searle’s directives) prompt actions in others;

Declaratives (correspond to Searle’s assertives) represent how things are.

Commissives (correspond to Searle’s commissives) refer to committing the speaker to future actions.

The four classes of communicative moves, rather than constituting a rigid taxonomy, provide a dynamic framework of social motives, a criterion that has so far been lacking in the study of emotional expressions. This very continuum of emotional expression also provides an explanatory framework for the performativity of stage directions. Stage directions generate communicative effects via emotion upon recipients in specific contexts, thereby contributing to the performativity of playtexts on stage. This, in turn, underscores the significance of affective theory in drama translation.

## 4 Research design

### 4.1 Research questions

To examine how a successful drama translation reconfigures stage directions expressing emotions to actualize performativity, this paper is guided by the following key research questions:

What strategies does Hong Shen’s translation adopt in rendering stage directions expressing emotions, in comparison with other Chinese translations? Do these strategies exhibit discernible regularities, and what effects do they produce?

How do the reconfigured stage directions in Hong’s translation perform different types of communicative moves in a specific context? In what ways do these reconfigured stage directions constitute a pre-performing space that mobilizes the performativity for the stage?

### 4.2 Research procedures

#### 4.2.1 Corpus and text processing

To address the research questions outlined above, the study builds an English-Chinese parallel corpus of Oscar Wilde’s *Lady Windermere’s Fan*. The corpus comprises approximately 143,354 tokens/characters (see Table 1 for details) and was aligned at the sentence level. The parallel corpus is available in the Supplementary material.

**TABLE 1:** English-Chinese parallel corpus of Oscar Wilde’s *Lady Windermere’s Fan.*

Source text/Translation	Author/Translator	Year	Source/Publisher	Tokens
*Lady Windermere’s Fan*	Oscar Wilde	1,893	The Bodley Head	41,133
*Yi Shan Ji* (遗扇记)	Shen Xingren (沈性仁)	1,918	*New Youth* (*La Jeunesse*)	32,920
*Shaonainai de Shanzi* (少奶奶的扇子)	Hong Shen (洪深)	1,925	The Commercial Press	34,993
*Wenfuren de Shanzi* (温夫人的扇子)	Yu Guangzhong (余光中)	1,983	Liaoning Education Press	34,308
Total				143,354

#### 4.2.2 Emotional word extraction based on NRC-EIL and DUTSD using Python

To analyze emotional expression as communicative devices, this study started by identifying emotional words based on Ekman’s theory, which provides probabilistic patterns in how language maps onto emotion. Building upon the methodologies of Kim and Klinger (2019) and Li et al. (2024), emotional words in source and target texts were extracted using the NRC Emotion Intensity Lexicon (NRC-EIL) and the Dalian University of Technology Sentiment Dictionary (DUTSD) (see Tables 2, 3). The full DUTSD lexicon is available in the Supplementary material.

**TABLE 2:** Emotional word distribution in NRC-EIL.

Emotion categories	Word numbers	Percentage
Anger	1,483	14.95%
Anticipation	864	8.71%
Disgust	1,094	11.03%
Fear	1,765	17.79%
Joy	1,268	12.78%
Sadness	1,298	13.08%
Surprise	585	5.90%
Trust	1,564	15.76%
Total	9,921	100%

**TABLE 3:** Emotional word classification in DUTSD.

Category	Sub-category	Category	Sub-category	Category	Sub-category
Happiness	Joy (PA)	Anger	Anger (NA)	Disgust	Boredom (NE)
	Relief (PE)	Sadness	Sorrow (NB)		Aversion (ND)
Appreciation	Respect (PD)		Disappointment (NJ)		Condemnation (NN)
	Praise (PH)		Guilt (NH)		Envy (NK)
	Trust (PG)		Longing (PF)		Suspicion (NL)
	Affection (PB)	Fear	Panic (NI)		
	Blessing (PK)		Dread (NC)		
Surprise	Surprise (PC)		Shame (NG)		

The extraction procedure followed a five-step workflow: (1) automated extraction of emotional words via a Python pipeline; (2) manual verification^4^, ^5^, ^6^, ^7^ with particular attention to ambiguity; (3) comparison of the two sets; (4) iterative refinement based on the comparisons. To assess the performance of the automatic extraction pipeline, evaluation metrics (Kumar and Kumar, 2025) were introduced as follows in this section.


Precision=5TPTP+FP

Recall=6TPTP+FN

F1-Score=72*⁢(Precision*⁢Recall)Precision+Recall

The performance (Precision, Recall, and F1-Score) of emotion identification based on the NRC-EIL dataset is 0.975, 0.73, and 0.835, respectively; that based on the DUTSD dataset is 0.893, 0.714, and 0.794. Besides, emotional intensity and polarity values were automatically assigned to each entry by the DUTSD dataset,^8^ which comprises 27,552 lexical entries with fine-grained granularity. Yet there still remain highly context-dependent entries or entries not covered by the dataset. Human annotators therefore re-evaluated each entry on a case-by-case basis with reference to its synonyms or antonyms. The annotation criteria and examples are outlined below (see Table 4).^9^

**TABLE 4:** Annotation criteria and examples.

Type of special cases	Example in Hong’s translation	Synonym or antonym in the DUTSD dateset	Final annotation (category, intensity, polarity)
Items with translator-specific style	瞪目结舌[stare in amazement]	瞠目结舌(as synonym)	PC, 9, 0
Culture-specific items	万福[curtsy]	鞠躬(as synonym)	PH, 1, 3
Items with intensifiers in context	十分感动[deeply moved]	深受感动(as synonym)	PA, 1, 3
Items with negation in context	不动怒[without anger]	动怒(as antonym)	NA, 1, 0
Context-dependent items	强笑[force a smile]	强颜欢笑(as synonym)	NB, 1, -1

#### 4.2.3 Investigation of Hong’s emotional reconfiguration strategies

Given the documented theatrical success of Hong Shen’s version, this section, first, conducted a translation method analysis based on Izwaini and Al-Omar (2019), categorizing methods into correspondence, adaptation, omission, and addition^10^ with an inter-coder reliability (Cohen’s Kappa) of 0.92.

Second, these methods were further encoded into granular emotional processing techniques, which were subsequently correlated with categories of stage directions to identify underlying patterns. To examine this correlation, a Chi-square test of independence was employed, as both variables are nominal (categorical). The key output statistics were interpreted as follows: χ^2^ measures the overall discrepancy between observed and expected frequencies, with larger values indicating a stronger association; the *p*-value indicates the probability of obtaining the observed χ^2^ under the null hypothesis of independence, and a value below 0.05 permits rejection of the null hypothesis at the conventional significance level; and Cramér’s *V* quantifies the strength of the association on a scale ranging from 0 to 1, with values above 0.5 generally considered a large effect size (Cohen, 1988).

Third, the study analyzed characters’ emotional features after Hong’s emotional reconfiguration, compared with the English original. A suite of indicators was employed as follows.

(1) Emotional density (emo_density) is defined as the quantitative ratio of a specific character’s emotional word count in stage directions to the number of times the character appears in the playtext (excluding any occurrences in dialogue). A higher density provides actors and directors with more frequent performative triggers.

(2) Mean valence (mean_val) is then calculated as the average product of polarity and intensity, often used as an indicator of the overall sentiment orientation. The formula is:


$$\text{mean\_val}=\frac{1}{n}\,\sum_{i=1}^{n}\left({\text{polarity}}_{i}\times {\text{intensity}}_{i}\right)$$

where polarity_*i*_ represents the emotional polarity of the *i*-th word (1 for positive, -1 for negative, and 0 for neutral); intensity_*i*_ denotes the emotional intensity of the *i*-th word, drawn from a five-level scale (1, 3, 5, 7 or 9); *n* is the total number of emotional words.

(3) Standard deviation (std_val) and valence range (range_val) are used to reflect emotional volatility, using the formula:


$$\text{std\_val}=\sqrt{\frac{1}{n}\,\sum_{i=1}^{n}{\left({\text{valance}}_{i}-\mathrm{mean}\,\text{\_val}\right)}^{2}},$$

$$\text{range\_val}=\max\left({\text{valance}}_{i}\right)-\min\left({\text{valance}}_{i}\right)$$

where valence_*i*_ is calculated as the product of polarity and intensity of the *i*-th word.

These indicators not only reflect the outcomes of Hong’s emotional reconfiguration strategies but also suggest a propensity for expressing emotions and enacting actions.

#### 4.2.4 Operationalizing communicative moves of emotional expression in stage directions

To analyze how emotional expressions produce performativity in drama translation based on Scarantino’s affective pragmatics, we make the following two adjustments to the theory. Firstly, in the original theory of affective pragmatics, the repeated categorization from “expressive moves” to “expressives” merely serves to emphasize the expressive function of nonverbal behaviors. Yet this emphasis is redundant, as the theory already presupposes that the nonverbal behaviors possess emotion at their very foundation. Precisely for this reason, we have from the outset extracted emotional words from stage directions. Therefore, we do not replicate this duplication, and retain only three categories of communicative moves for emotional expression: declaratives, imperatives, and commissives.

In this study, all stage directions are classified into four types: psychological, prosodic, facial, and kinesic stage directions. Declaratives correspond to psychological stage directions that explicitly reveal the internal mental state of the character; imperatives correspond to kinesic, prosodic, and facial stage directions manifested as directives for specific actions; commissives are interpreted in context, without being strictly confined to a fixed category. Given that all communicative moves of emotional expression are proposed based on Austin’s performative utterances, this classification inherently reflects their performative orientation.

These three categories are not discrete but rather form a continuum of mutual integration and transformation depending on the historical and cultural context in which they occur. In a playtext, kinesic stage directions like a bow expressing respect act as explicit requests to actors as imperatives; while an apologetic bow also functions as commissives, implicitly promising to make amends, and a ritual bow at a wedding vow to fulfill familial duties. This fluidity has direct implications for drama translation: how a drama translator handles stage directions shapes the performativity of emotional expression on stage.

## 5 Results

This section begins with an overview of Hong’s translation methods, followed by a detailed investigation into emotional processing techniques in order to uncover underlying patterns, and culminates in evaluating the effects of Hong’s reconfiguration strategies.

### 5.1 Comparative analysis of translation methods

A pronounced divergence in translation methods emerges. While Shen’s and Yu’s versions demonstrate a marked preference for direct correspondence, Hong’s translation is characterized by additions (153 instances), adaptations (40 instances), and omissions (33 instances). Hong’s strategic shift toward creative reinterpretation stands in stark contrast to the literary fidelity prioritized in Shen’s and Yu’s versions, thereby providing compelling empirical evidence for a fundamental paradigm shift in drama translation.

### 5.2 Investigation into emotional processing techniques

Building upon the macro-analysis, this section further examines specific emotional processing techniques employed across three Chinese translations. Regarding omissions, Shen’s version omits eight stage directions (e.g., *smiling*, *bowing*, *interrupting*, *frowning*), while Yu’s version omits only three prosodic stage directions related to pauses. In stark contrast, Hong omits 33 instances, exhibiting a strategic pattern: by types, these primarily involve 15 psychological and 8 prosodic (mostly pauses) stage directions; by characters, the focus is squarely on Lady Windermere (11 instances) and Mrs. Erlynne (8 instances).

Regarding additions, while two other versions contain no additions, Hong’s translation includes 153 additions, with 79 psychological stage directions, 39 facial cues, 24 kinesic cues, and 11 prosodic cues. This distribution shows that Hong prioritizes the rendering of inner psychological landscapes, while also substantially supplementing visual and vocal elements to enhance the performativity of the translated text on stage.

Regarding adaptation. Shen’s version contains only 4 instances. For example, the kinesic stage direction “smooth his hair” is rendered as “抓耳挠腮” (scratching one’s head and ears). This represents minor pragmatic adjustments within a broader strategy of literal fidelity. While Yu’s version contains no adaptations, Hong’s version includes 42 adaptations, which can be further summarized into three strategies: type transformation (converting types of stage directions), content adaptation (altering descriptions within the same type), and descriptive expansion (deepening existing textual cues).

To further explore underlying patterns of emotional processing techniques in Hong’s version, we conducted a correlation analysis between techniques and stage direction types. The Chi-square test indicates that while Hong’s techniques exhibit a remarkably strong significant correlation with stage direction types (χ^2^ = 90.68, *p* = 0 < 0.05, *V* = 0.85>0.5), manifested in four primary aspects.

First, regarding psychological stage directions, Hong frequently retains the original type of stage directions but employs semantic reinforcement to intensify and clarify emotion, such as adapting “hesitate” in the source text into “言外似乎有意” (seemingly carry an overtone) or “难为” (finding it difficult); notably, Hong often converts these psychological cues into prosodic, kinesic, or facial stage directions, externalizing inner states as physical movements.

Second, kinesic stage directions are frequently refined for greater precision. For instance, a simple “bow” may be augmented with adverbs or adjectives to “略一鞠躬” (a slight bow) or “低低的鞠躬” (a low bow)—thereby breaking down and layering indexes to strengthen stage practicality and cultural resonance.

Third, prosodic stage directions are primarily converted to facial expressions: “in a low voice” is transformed into more visual cues, such as “脸上做出许多怪相” (making strange faces) or “鬼鬼祟祟” (sneaking). Finally, facial stage directions are expanded or altered to make emotion explicit, as seen when “frown” is expanded to “觉得刺耳, 皱了眉头” (feeling ear-piercing, he frowned), effectively shifting the internal state into a visible, dramatic expression.

### 5.3 Evaluation of the emotional reconfiguration effect

In this section, the study calculates emotional density, mean valence, valence standard deviation, and valence range for characters in both the English source text and Hong’s translation (see Table 5), to reflect general trends in emotional polarity and emotional fluctuations across characters in Hong’s version. The other two versions are excluded from this in-depth affective analysis due to their high degree of literal correspondence to the source text.

**TABLE 5:** Emotional reconfiguration effect of source text and Hong’s translation.

English source text					Hong’s translation				
Character	emo_ density	mean_ val	std_val	ran_val	Character	emo_ density	mean_ val	std_val	ran_val
Full text	0.0028	−0.975	4.48	18	Full text	0.0057	−0.68	4.63	18
Lady Windermere	0.13	−2.24	4.59	16	Lady Windermere	0.27	−0.66	4.80	18
Mrs. Erlynne	0.23	−0.61	5.11	16	Mrs. Erlynne	0.31	−0.55	5.07	18
Lord Windermere	0.11	−1.89	4.97	16	Lord Windermere	0.23	−1.09	4.59	18
Lord Augustus	0.13	0.33	5.43	14	Lord Darlington	0.2	−0.35	4.41	14
Cecil Graham	0.14	−0.11	4.67	14	Cecil Graham	0.23	−0.21	3.90	8
Lord Darlington	0.05	0.88	3.56	10	Duchess of Berwick	0.2	−0.50	4.50	10
Dumby	0.10	3.5	5.45	18	Dumby	0.12	−0.25	3.65	10
Duchess of Berwick	0.08	0	5.66	10	Lord Augustus	0.15	0.64	4.39	12
Hopper	0.07	5	0	0	ShiYe^1^	/	5.00	2.83	6
Lady Jedburgh	0.09	3	0	0	Hopper	0.15	2.33	1.15	2

^1^Hong’s translation adds a character, *Shi Ye* (a legal advisor), who does not appear in the source text.

As shown in Table 5, the source text generally exhibits a neutral-to-negative emotional orientation, reflecting the play’s inherent critical tone, while std_val shows a high level of emotional fluctuation. At the full-text aggregate level, the net differences between Hong’s translation and the source text appear marginal. However, these global averages may mask substantial character-level heterogeneity. To address this, we conducted a character-level paired analysis (*N* = 9) with permutation testing (10,000 iterations) and computed the 95% confidence interval (CI) for each metric.^11^ Three principal patterns emerge from this analysis.

First, translation yields a statistically significant increase in emotional density at the character level [Δemo_density = +0.091, 95% CI: (0.055, 0.128), *p* < 0.01], confirming that systematic addition techniques result in a more affectively saturated text. Second, the translation exhibits an overall shift toward neutrality [Δmean_val = −0.611, 95% CI: (−1.91, 0.69), *p* = 0.32>0.05], though this aggregate shift is non-significant and character-dependent. While the elevated mean valence partially offsets the source text’s pervasive negativity, a character-specific divergence emerges: mean valence increases for Lady Windermere, Mrs. Erlynne, Lord Windermere, and Lord Augustus, but decreases for Lord Darlington and others. This pattern suggests that Hong amplifies positive emotions for core characters while accentuating negative ones for others, thereby constructing a binary emotional narration. Third, the translation demonstrates a trend toward heightened emotional volatility, though the overall rise in standard deviation is not statistically robust [Δstd_val = −0.331, 95% CI: (−1.08, 0.41), *p* = 0.41>0.05]. Notably, this intensification is strategically concentrated. The standard deviation increases for Lady Windermere and Lord Darlington while leveling off for others, effectively focusing emotional tension on the plotlines of these two characters. Crucially, as the narrative hub, Lady Windermere’s reinforced positive valence and increased fluctuation serve as key indicators of how Hong leverages emotional reconfiguration to maintain internal character consistency while maximizing dramatic tension.

## 6 Discussion

### 6.1 Emotional expression as declaratives

Declaratives have the capacity to represent how things are in the world, though there is no consensus on what makes emotions representational in the first place (Scarantino, 2017, p. 176). Emotional memory, in which actors draw on their own past real emotional experiences, explains why textual emotional markers can be performed: it triggers the actor’s internalized emotions in given circumstances (Stanislavski, 2022). Hong Shen added 79 psychological stage directions, omitted 15, and also incorporated supplementary psychological details when rendering other types of stage directions. In this way, he made the characters’ inner states more explicit, as he perceived them.

In Example 1, Hong adapted the original gestic cues into a series of psychological cues, constructing a clear emotional conflict: the character was caught between desire and social propriety, at this moment trying to grasp a transient chance. The more specific the description is, the easier it is for the actor to relate to similar experiences, thereby activating corresponding emotional memory and achieving an internalized emotional expression.

Example 1:

Source text: LORD DARLINGTON. Ah, but I did mean them. (Takes tea which she offers him.)

Hong’s translation: 伯英：（他对徐夫人，确存非分之想；但言谈举止，尚不敢大，胆放肆。）此刻好像有机可乘，再也不肯放过。 [(Lord Darlington He does love Mrs. Xu, though he has not yet been bold enough to show his love. Now, seeing a possible chance, he will not let it pass.) I am perfectly honest, and I mean every word I say.]

It should be noted that Hong’s pioneering strategies resonate across time and space with the concept of “writing forward” proposed by Spanish scholar, playwright, and translator David Johnston. Writing forward refers to “bringing the potentials for performance that are encoded in the original explicitly into the temporal and spatial purview of the audience” (Johnston, 2013, p. 366), thereby projecting the impact of that context into the emotional landscape of the new audience, potentially generating a sort of temporal interface, a crossing point between core experiences lost and, hopefully, re-created (Johnston, 2015, p. 20). Consequently, whether the playtext serves as a vehicle for surtitles in international productions (Marinetti, 2013) or as one of the elements of the dramatic spectacle (Ladouceur, 2013; Nolette, 2014), its semiotic value remains paramount across multiple contexts (Morini, 2022, p. 46).

### 6.2 Emotional expression as imperatives

The imperative function of emotional expression is to elicit acts by recipients. Such bodily mobilization is also treated as the synonym with doability (Elam, 1980) in drama theory, or arousal (Bradley and Lang, 2007) in emotion theory. On a closer look, emotional expression with a higher arousal entails higher intensity and involves more body mobilization (Scarantino, 2023, p. 606). Such high arousal is a distinctive feature of Hong’s translation, for he, while preserving the basic content of the source text (with only 8 omissions), added 74 embodied stage directions, that is, facial, kinesic, and prosodic ones, and also rewrote a large number of psychological stage directions into embodied ones. This is why the emotional density and intensity of stage directions in Hong’s translation are generally enhanced after his emotional reconfiguration.

In Example 2, Hong amplified the original cues by adding a series of kinesic and psychological stage directions that create a symmetrical interplay between characters. And the idiom “shrinking from a snake or scorpion” replaces the declarative expression “contempt,” conveying dread and disgust simultaneously. Hong’s version transforms a psychological state into a dynamically performable action, making the character’s inner repulsion tangible to both actors and readers. More broadly, his translation excavates the affective potential of stage directions, treating them not merely as functional cues but as a pre-performing space. This approach moves beyond faithful linguistic conversion toward an emotional reconfiguration, highlighting performativity toward the stage.

Example 2:

Source text: MRS. ERLYNNE. Lady Windermere!

LADY WINDERMERE. [startles and looks up. Then recoils in contempt.]

Hong’s translation: 金女士:（匆匆走入, 开灯寻见徐夫人, 又惊又喜。）

奶奶: （听唤吃惊, 慢慢退缩, 如避蛇蝎) [Mrs. Erlynne: (Hurries in, switches on the light, sees Mrs. Xu, and is both startled and overjoyed.) Mrs. Xu! Lady Windermere: (Starts at the call, then slowly recoils, as if shrinking from a snake or scorpion).]

What Hong performs in his translation is precisely a move from textual fidelity to performative reinvention, where the drama translator emerges as both co-subject and co-author of the performance, competent both in textuality and performativity for stage as well as acting conventions (Bigliazzi et al., 2013, p. 13). This shift highlights the translator’s autonomy as a creator of the target text and accentuates the performativity dimension of drama translation (Morini, 2022, p. 47).

### 6.3 Emotional expression as commissives

Commissives commit the sender to a future course of action by means of natural information transfer (Scarantino, 2017, p. 176). A retrospective of theater history reveals that while the relationship between dialogue and stage directions varies across eras (Ubersfeld, 1999, p. 8), these directions, once woven into the textual fabric, function as indispensable emotional carriers, that anchor prospective commitments, making them crucial components of the emotional narrative.

In Example 3, Lord Darlington harbored romantic love for Lady Windermere, who was already married. So he suppressed his affections and withdrew from her life. In Hong’s translation, the kinesic stage direction “exit” was adapted into a chain of cues, tracing the character’s emotional trajectory from inner turmoil to final resolution. The deep bow, in particular, functions on multiple levels: it not only symbolizes social courtesy but also constitutes a ritualized gesture of farewell, marking a regretful end to the relationship. This commissive emotional expression, in turn, generates a narrative contrast with the later scene where Lady Windermere stole into Lord Darlington’s house, thereby heightening the emotional tension.

Example 3:

Source text: LORD DAR They must never meet to touch again. Good-bye, Margaret. [Exit.]

Hong’s translation: 伯英: 以后这两条心, 总不要再接近罢。（要走, 回转头, 来又看了徐夫人一会，深深鞠躬一径走了。） [ Lord Darlington From now on, let these two hearts never draw near each other again. (He is about to leave, but turns back, gazes at Mrs. Xu for a moment, makes a deep bow, and then walks straight away.)]

From plot design to performance-oriented stage directions, Hong’s adaptation highlights his identity across multiple stages of theatrical production. Such a collaborative essence reflected in Hongshen’s version is also underscored by Johnston’s idea of writing forward. As a pioneer of modern Chinese drama, Hong Shen integrated his experience as a director of classics such as “法西斯细菌” (Fascist Bacteria) and “草莽英雄” (The Hero of the Wilds), a playwright of works such as “赵阎王” (Yama Zhao) and “贫民惨剧” (The Tragedy of the Poor). Furthermore, he actively introduced Western drama by translating The Emperor Jones and authored the seminal theoretical work, “电影戏剧表演术” (The Art of Film and Drama Performance). This synergy of roles, spanning creative practice, theoretical inquiry, and the introduction of Western drama—has forged a unique translation paradigm that privileges the stage as the ultimate field and performable literariness as the core.

## 7 Conclusion

Through multi-layered analysis, this study finds that both Shen’s and Yu’s translations of stage directions reproduce the original emotional expression through faithful rendering. In contrast, Hong Shen employs translation methods varying across stage direction types through addition, adaptation, and omission. Specifically, Hong adds chains of psychological stage directions supplemented with detailed descriptions, while converting relatively static psychological descriptions into dynamic kinesic, prosodic and facial cues that enact emotions. Such emotional reconfiguration strategies ultimately recreate emotional narration distinct from the source text, characterized by increased emotional density, intensified conflict with a shift toward neutrality, polarized emotional intensity, and amplified emotional fluctuations.

Underpinning this reconfiguration are three communicative moves of emotional expressions: declaratives prompt explicitation by clarifying contextual presuppositions; imperatives invite embodiment by translating verbal orders into gestic cues; and commissives generate plot propulsion by anchoring characters’ future actions. Together, these functional mappings reconfigure the source text’s performativity across verbal, physical, and temporal dimensions. As Freytag (1990, p. 10) noted, the essence of dramatic art lies not merely in presenting events, but in revealing the emotions that trigger actions and the impact of those actions on the soul.

Echoing the writing forward strategy proposed by drama theorist David Johnston, Hong Shen’s practices essentially interrogate the propositions of translation ethics and limits. In a globalized context where traditional notions of fidelity are challenged by AI translation, the unique value of the drama translator as a collaborator in stage art lies in transcending superficial linguistic conversion. They must elevate drama translation into a performative practice that integrates stage experience with cultural insight, seeking an irreplaceable foundation in the AI era. This case study of a successful translation reveals the recreation essence of drama translation. And the multi-modal cognitive advantages of a translator with multiple identities like Hong Shen also provide a practical paradigm for resolving the binary opposition between readability and performability, offering methodological enlightenment for contemporary drama translation practice.

## Data Availability

The original contributions presented in the study are included in the article/Supplementary material, further inquiries can be directed to the corresponding author.

## Footnotes

1 The version analyzed in this study is Hong Shen’s 1923 translation as compiled in the *Collected Works of Hong Shen (Vol. 1)* (1988). It should be noted that this is the published edition rather than the script used in theatrical productions.

2 Founded in 1921, the Shanghai Stage Society (Shanghai Xiju Xieshe) was a pioneering amateur troupe; in 1923 Hong Shen joined as its rehearsal director, and his adaptation of Oscar Wilde’s *Lady Windermere’s Fan* became a landmark success that established modern directing practices and mixed-gender casting in Chinese theater.

3 Charles Sanders Peirce’s triadic model of signs posits that every sign consists of three interrelated elements: the Representamen (the sign itself), the Object (that which the sign stands for), and the Interpretant (the effect or understanding generated in the mind of the interpreter) (Peirce, 1960, p. 285). Building upon this framework, semiosis refers to the dynamic and recursive process in which a sign generates an interpretant, which in turn becomes a new sign, thereby initiating an endless chain of interpretation (Peirce, 1960, p. 141).

4 Two trained annotators independently classified each entry using the predefined criteria. To minimize cognitive biases, a structured reconciliation protocol was implemented: annotation, cross-examination, discussion, agreement. All discrepancies underwent iterative discussion: annotators cross-examined contested cases and refined classifications through mutual debate until full agreement.

5 Precision measures the exactness of the extraction. True Positives (TP) refer to the number of emotional words that are both extracted by the machine and marked by human annotators; False Positives (FP) refer to the number of words extracted by the machine but not marked by human annotators; False Negatives (FN) refer to the number of words that are not extracted by the machine but marked by human annotators.

6 Recall measures the completeness of the extraction.

7 The F1-Score is a harmonic mean of precision and recall, which is a balance indicator for the accuracy of the model.

8 The emotional intensity is rated on a five-point scale (1, 3, 5, 7, and 9), while polarity is represented by three values: 1 for positive, 0 for neutral, and -1 for negative.

9 It should be noted that words that carry no emotional connotations in the context of this text were also excluded. For instance, the term 后台 *hòutái* refers literally to backstage in translation and carries no emotional bias, which differs from its metaphorical annotation in the DUTSD, that is, a power, figure, or organization that provides support for someone or an activity behind the scenes.

10 Correspondence refers to the method of employing a target-language equivalent that closely matches the source text in both form and meaning, typically realized through literal or word-for-word translation; adaptation involves adjusting the source text to conform to the norms of the target language, this may entail restructuring or cultural alternatives while the core content is preserved; omission is the deliberate exclusion of certain elements from the source text; addition, conversely, is the supplementation of explicit information in the target text, including connectives, clarifications, or cultural notes.

11 The *p*-value tests the null hypothesis of no translational effect, with *p* < 0.05 indicating statistical significance. The 95% CI provides a range of plausible effect magnitudes: if the interval excludes 0, the effect is significant at the α = 0.05 level; if it includes 0, the direction of the effect remains inconclusive.
